# Practical implications to contemplate when considering radical therapy for stage III non-small-cell lung cancer

**DOI:** 10.1038/s41416-020-01072-4

**Published:** 2020-12-08

**Authors:** Claire L. Storey, Gerard G. Hanna, Alastair Greystoke, on behalf of AstraZeneca UK Limited

**Affiliations:** 1 Newcastle upon Tyne Hospitals NHS Foundation Trust, Newcastle upon Tyne, UK; 2 Patrick G. Johnston Centre for Cancer Research, Queen’s University Belfast, Belfast, UK; 3 Sir Peter MacCallum Department of Oncology, University of Melbourne, Melbourne, Australia; 4 Newcastle University Centre for Cancer, Newcastle University, Newcastle upon Tyne, UK

## Abstract

The type of patients with stage III non-small-cell lung cancer (NSCLC) selected for concurrent chemoradiotherapy (cCRT) varies between and within countries, with higher-volume centres treating patients with more co-morbidities and higher-stage disease. However, in spite of these disease characteristics, these patients have improved overall survival, suggesting that there are additional approaches that should be optimised and potentially standardised. This paper aims to review the current knowledge and best practices surrounding treatment for patients eligible for cCRT. Initially, this includes timely acquisition of the full diagnostic workup for the multidisciplinary team to comprehensively assess a patient for treatment, as well as imaging scans, patient history, lung function and genetic tests. Such information can provide prognostic information on how a patient will tolerate their cCRT regimen, and to perhaps limit the use of additional supportive care, such as steroids, which could impact on further treatments, such as immunotherapy. Furthermore, knowledge of the safety profile of individual double-platinum chemotherapy regimens and the technological advances in radiotherapy could aid in optimising patients for cCRT treatment, improving its efficacy whilst minimising its toxicities. Finally, providing patients with preparatory and ongoing support with input from dieticians, palliative care professionals, respiratory and care-of-the-elderly physicians during treatment may also help in more effective treatment delivery, allowing patients to achieve the maximum potential from their treatments.

## Background

The standard curative-intent treatment for patients with unresectable stage III non-small-cell lung cancer (NSCLC) is concurrent chemoradiotherapy (cCRT).^1,2^ The use of cCRT is associated with improved survival when compared with sequential chemoradiotherapy (sCRT), although at the cost of increased toxicity.^3^ Rates of cCRT vary between, and within, countries. Higher-volume centres will treat patients with more co-morbidities and higher-stage disease; however, in spite of these disease characteristics, patients have improved overall survival (OS).^4^ This suggests that improvements in treatment allocation and delivery could lead to improved survival.

Patient selection remains key. In systematic reviews of NSCLC clinical trial data, a 1–3% mortality rate has been reported; however, this is dependent on the trials included in these analyses and the exact definition of death whilst on treatment, and how it is attributed.^5,6^ The most common cause of treatment-related death was radiation pneumonitis (accounting for 33.2% of deaths in one series) with neutropenia, pneumonia, haemorrhage, infection, acute respiratory distress syndrome and cardiac disease as other frequent causes of death related to treatment.^7^

Most patients diagnosed with NSCLC are either current or ex-smokers and frequently have other co-morbidities, including chronic obstructive airway disease, coronary artery disease or other peripheral vascular disease, which may be a predictive factor for increased toxicity during treatment.^8,9^ In a review of 577 patients presenting with stage III NSCLC in The Netherlands between 2002 and 2005, the proportion of patients who had one or more serious co-morbidities, or who were >75 years of age, was assessed. If these criteria are used to exclude patients from cCRT, then 59% of this study group would be ineligible.^10^

Comprehensive guidelines for the assessment of patients’ fitness prior to chemoradiotherapy (CRT) do not exist. In 2009, the European Respiratory Society and European Society of Thoracic Surgeons published clinical guidelines on the fitness for lung cancer patients undergoing radical therapy, but these concentrated primarily on fitness for surgery. The group did not feel that it was possible to produce guidelines for lung cancer patients being initiated on CRT due to the lack of supporting evidence.^11^

If the strict inclusion and exclusion criteria used in clinical trials are used in the clinic, patients may be unnecessarily excluded from their optimal chance of receiving curative treatment. This is increasingly the situation, given the improvements in the delivery of both chemotherapy and, in particular, radiotherapy over the last 10 years, which may lead to substantially lower rates of toxicity.

In spite of the current evidence gaps, and with the aim of ensuring safe delivery of cCRT in the treatment of stage III NSCLC, here we aim to set out the current knowledge and best practice to optimally (a) assess a patient for treatment, (b) deliver chemotherapy concurrently, (c) deliver radiotherapy in the concurrent setting and (d) provide supportive care for patients receiving CRT.

## Patient assessment for chemoradiotherapy

### Disease-staging assessments

Prior to delivery of concurrent chemoradiotherapy, it is important to ensure that the patient has received all appropriate staging investigations. Whilst many of the original clinical trials of CRT were performed prior to the routine availability of positron emission tomography (PET), this along with cross-sectional imaging of the brain using either computerised tomography (CT) or magnetic resonance imaging (MRI) should now be regarded as standard for the diagnosis and staging of NSCLC patients being considered for radical therapy.^1^ Additional imaging procedures, such as endoscopic bronchial ultrasound (EBUS) and mediastinoscopy, can also be used to aid diagnosis.^1^ This will help to ensure that only patients with localised disease, with a chance of cure, receive cCRT and thus avoid unnecessary toxicity. Whether this combined modality treatment has a role in the oligometastatic setting is currently under investigation in clinical trials such as Stereotactic Ablative Radiotherapy for Oligometastatic Non-small Cell Lung Cancer (SARON).^12^

### Patient performance status

Performance status (PS) remains a key assessment criterion. In clinical trials reporting outcomes for CRT, the vast majority of patients were of Eastern Cooperative Oncology Group (ECOG) PS 0–1, with only 2% of patients with an ECOG PS of 2 being included.^3^ It is likely that patients with a poorer functional status will struggle to complete the treatment course, resulting in inferior outcomes and possibly being unable to receive a ‘curative’ dose of radiotherapy. Assessment of reversible components of PS should be made, especially if reduced PS is a result of incurrent infection, poor control of airway disease or uncontrolled pain. However, even if corrected, these patients may be at risk of increased toxicity. In patients with PS ≥ 2, consideration of alternative treatment strategies should be made, including sCRT, radiotherapy alone or active symptom control, depending on the extent of functional impairment and the patient’s wishes.

### Impact of patient age

Data on the safety and efficacy of CRT in older patients are lacking. For example, only 13% of patients were 70 years or older in the concurrent treatment arms in the meta-analysis reported by Auperin et al.^3^ There is variability in treatment, with a review of the SEER database showing that only 36% of patients over 65 years presenting with stage IIIA NSCLC receive CRT, with substantial variation seen throughout the United States.^13^ There may be reluctance to treat older patients even in the absence of comorbidity or poor PS.^14^ In a pooled analysis of individual patient data on cCRT for stage III NSCLC in patients aged over 70 years who participated in US National Cancer Institute Cooperative Group studies, older patients experienced worse survival outcomes, increased toxicity and a higher rate of treatment-related death than younger patients.^15^ In another review that included 216 patients over 70 years old in The Netherlands, co-morbidities, PS (or a combination of both of these, 57%) and patient refusal (15%) were the most common reasons for not undertaking cCRT.^8^ Comorbidity was associated with toxicity in patients receiving both concurrent and sequential CRT.^8^ Although difficult to deliver routinely to all cancer patients, Comprehensive Geriatric Assessment may have value in patients over 75 being considered for cCRT. In a recent publication of 85 patients over 75 with stage III NSCLC, 37% of patients were rated as fit (no disability on the activities of daily living [ADL] or instrumental activities of daily living [IADL] scales, comorbidity score <2) and 48% as medium-fit (<3 IADL, no ADL disability, comorbidity score <3).^16^ Higher scores on the Vulnerable Elders Survey were associated with shorter survival and a higher risk of grade 3–4 toxicity.^16^

### Risk factors predictive of radiation pneumonitis and acute oesophagitis

The risk of radiation pneumonitis and acute oesophagitis are the primary normal tissue toxicity considerations that limit treatment delivery. Both patient and tumour factors may help predict the risk. Models have been published, which assess the risk of pneumonitis. One investigation looked at data from 438 patients receiving thoracic radiotherapy to determine which patient characteristics were predictive factors for radiation-induced pneumonitis. Assessed factors included PS, smoking status, forced expiratory volume in one second (FEV1), age and mean lung dose (MLD). The strongest prognostic factor for pneumonitis was FEV1, with an odds ratio of 0.98 (95% CI 0.97–0.995, *P* = 0.004) with other significant factors in the model being PS, smoking status, age and MLD.^17^ FEV1 is often reported as a percentage of normal value, corrected for patient factors; FEV1 assessment can become problematic at extremes of age and in female patients with a lower body mass index. It is also affected by day-to-day fluctuations in respiratory symptoms, and can often depend on operator skill to achieve a reproducible and optimal reading.^18^

Alongside the assessment of airway spirometry, diffusion-capacity measurements as a pre-treatment assessment are considered good practice when determining surgical treatment of less-advanced lung cancers.^11^ This measurement is also useful in the pre-treatment assessment of patients with more advanced lung cancers as it may allow clinicians to identify patients who will not tolerate complications such as pneumonitis. It may also allow diagnosis of the underlying lung disorders such as pulmonary fibrosis, which increases the risks of complications and potentially changes the available treatment options for the patient to maintain an acceptable quality of life without unnecessary toxicity.^19^

Using published data, the STRIPE project analysed individual patients who had received cCRT in an attempt to identify factors predictive of pneumonitis. After randomly dividing patients into a training and validation group, and using recursive partitioning analysis (RPA), the authors identified age of over 65 years and receiving carboplatin paclitaxel chemotherapy as factors most predictive of symptomatic pneumonitis, and receiving a hypofractionated regimen (daily radiotherapy dose >2 Gy), the volume of lung receiving 20 Gy (V20) and lower-lobe tumour location were all predictors of fatal pneumonitis.^20^ However, the authors did not evaluate smoking history, details of co-morbidities or pulmonary function testing. Other clinical studies have also indicated V20 as a predictor of pneumonitis in stage III NSCLC, including a multivariate analysis which reported that a V20 greater than or equal to 26% was an independent risk factor.^21^ Studies such as these have led to suggestions from organisation bodies, for example, the European Organization for Research and Treatment of Cancer (EORTC), that if possible, the V20 and the mean lung dose should be kept at 35–37% and 20–23 Gy, respectively, but that these criteria were not limiting, and could require a full diagnostic assessment of the patient by an expert respiratory physician.^22^

**Fig. 1:**
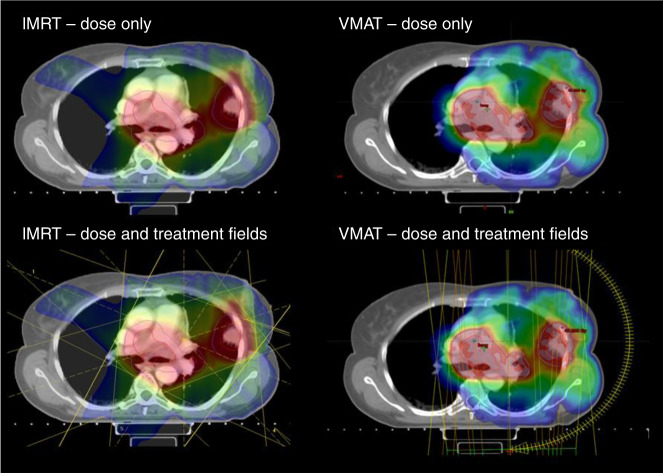
Intensity-modulated radiotherapy (IMRT) and volumetric arc radiotherapy (VMAT) plans are shown for the same patient with stage III non-small-cell lung cancer (NSCLC). Illustrated in these figures are the differences in radiotherapy dose distribution to the lungs following IMRT or VMAT. These images show that using advanced IMRT may help to decrease the V20 dosimetric value to the non-target lung, but may also increase the proportion of lung receiving lower doses of radiotherapy than when using VMAT. The threshold dose for the colour wash used is 18 Gy. On the left-hand side, the dose distribution and field arrangements for a fixed-field IMRT plan are shown. On the right-hand side, dose distribution and field arrangements for the VMAT plan are shown. Through the use of advanced IMRT technique, VMAT sparing of the contralateral lung is possible, and this is illustrated by the absence of any significant dose above 18 Gy in the right lung on the VMAT plan, as compared with the IMRT plan. Images provided by authors.

Whilst there are little published data on the risk in patients with underlying fibrotic or interstitial lung disease (ILD) in locally advanced NSCLC, in early-stage disease, the presence of pre-existing ILD increases the risk of pneumonitis,^19^ and expert opinion is that the presence of ILD is associated with an increased risk of lung toxicity.^22^ Improvements in the delivery of radiotherapy (e.g., the use of intensity-modulated radiotherapy [IMRT]) may help to decrease the V20 dosimetric value, but may also increase the proportion of lung receiving lower doses of radiotherapy (e.g., volume receiving 5 Gy (V5))^23^ as detailed below and in Fig. 1.

Rates of grade 3 oesophagitis between 1 and 18% have been reported following cCRT.^6^ Patient risk factors for oesophagitis include Caucasian race, age of ≥70 years, poor initial PS (≥ 2), low body mass index and gastro-oesophageal reflux.^24^ In terms of tumour factors, central location and nodal stage are associated with higher rates of oesophagitis because of the greater extent of the oesophagus irradiated and the higher doses delivered.^24^ An individual patient meta-analysis of 1082 patients undergoing CRT for locally advanced NSCLC (using the same RPA analysis method described above) reported that the oesophageal volume receiving ≥60 Gy (V60) alone was the best predictor of grade ≥2 radiation oesophagitis, with a V60 < 0.07% associated with less than 5% risk of grade ≥3 oesophagitis, and a V60 ≥ 17% conferring a 59% risk of grade ≥2 and 22% risk of grade ≥3 oesophagitis.^25^ The type of chemotherapy used may also affect the incidence of oesophagitis. De Ruysscher et al. reported that worse neutropenia during CRT was associated with worse dysphagia^26^, whilst different platinum doublets may impact on individual risks.

### Assessment of renal and cardiac function

Given the nephrotoxicity of platinum agents, in particular cisplatin, baseline assessment of renal function is required. Renal function may be improved by stopping nephrotoxic drugs such as non-steroidal anti-inflammatories and assessing patient requirement for anti-hypertensives they are currently receiving. The Cockcroft and Gault equation often underestimates glomerular filtration rate (GFR) in older patients and those with low muscle mass.^27^ If poor estimated renal function is a potential factor in a decision not to deliver cCRT, it should be formally assessed, for example, using the excretion of Chromium-51 EDTA. This is also vital if carboplatin is to be used to ensure the accuracy of dosing in this situation.

Although assessment of cardiac physiology is one of the main components of assessment for lung cancer surgery,^11^ it is not routinely used in chemoradiotherapy. Cardiopulmonary exercise testing has been used to document decreases in aerobic threshold in patients receiving CRT in other settings such as oesophageal cancer;^28^ however, its utility for patient selection and monitoring in NSCLC is largely unknown. In addition, the CRT dose to the heart may be an important and under-recognised problem with a significant impact on long-term health.^29^

### Additional patient considerations for chemoradiotherapy with consolidation immunotherapy

As the use of consolidation immunotherapy following cCRT is implemented, it will also be important to assess the patient’s fitness for this treatment modality. In the PACIFIC study, before being randomised to potential treatment with durvalumab, patients not only had to have a PS of 1 following the completion of cCRT, with documented response to treatment (i.e., stable disease), they also had to have no previous exposure to anti-PD-1 or PD-L1 antibodies, receipt of immunotherapy or an investigational drug within 4 weeks before the first dose.^30^ These included active or previous autoimmune disease within the last 2 years, with the exception of vitiligo, Graves’ disease and psoriasis not requiring systemic therapy.^31^ Whilst there is increasing experience of carefully treating selected patients with these co-morbidities and metastatic disease with immunotherapy agents,^32^ the safety of PD-L1 inhibitors in this indication has not yet been established. In addition, patients requiring more than 10 mg of prednisone a day (or an equivalent steroid dose) were excluded from the PACIFIC study.^31^ Although unlikely to impact on tolerability, there is increasing evidence from the metastatic setting that patients who require more than this dose of steroids prior to, or shortly after, initiating treatment with an immunotherapy, have poorer outcomes.^33^ This has yet to be assessed in the setting of stage III NSCLC patients treated with cCRT.

## Optimisation of chemotherapy in patients with stage III NSCLC

Although an initial study showed the benefit of adding single-agent cisplatin to radiotherapy for the treatment of inoperable NSCLC,^34^ current guidelines suggest the use of a platinum-based doublet chemotherapy in fit patients to maximise the chance of cure.^1^ The weekly regimen of carboplatin and paclitaxel has been used in a number of US-based clinical trials as the standard-of-care control arm;^35^ however, this has not been widely adopted in Europe. This may be an option for patients who are cisplatin-ineligible, for example, because of borderline renal function. Both guidelines^1^ and population-based studies suggest that if a carboplatin regimen is used during radiation, consolidation chemotherapy may be more important.^36^

The combination of chemotherapy with radiation impacts the adverse events (AEs) experienced, and therefore the choice of chemotherapy should account for the toxicity profile as well as efficacy. In the PROCLAIM study, patients randomised to cisplatin/pemetrexed had significantly less haematological toxicity than those receiving cisplatin/etoposide, with lower grade 3 or 4 AEs of neutropenia, febrile neutropenia and thrombocytopenia.^37^ Similarly, in a community review of 1842 patients treated within the Veterans Health Administration, patients receiving cisplatin and etoposide had more hospitalisations, infectious complications, renal complications and mucositis or oesophagitis than those receiving carboplatin and paclitaxel.^38^

## Optimisation of radiotherapy in patients with stage III NSCLC

Over the last decade, the clinical experience and technical delivery of external beam radiotherapy in the treatment of lung cancer has changed considerably, which has led to the ability to deliver curative-intent radiotherapy to larger tumours and patients with poorer fitness levels. The expertise of the clinical or radiation oncologist, clinical physicists team and radiographers has developed considerably through initiatives such as clinical trial quality assurance,^39^ colleague peer review of target volume delineation^40^ and education initiatives such as contouring workshops.^41^

Target volume delineation has improved in accuracy and reproducibility with the use of staging with PET-CT scanning and information from diagnostic procedures such as EBUS.^42,43^ The technical delivery of radiotherapy has evolved from using two- and three-dimensional planning techniques to using IMRT, which is superior at reducing radiotherapy dose to normal structures with no tumour involvement. Computerised radiotherapy planning systems now give superior estimations of the dose received by tumours in the lung through improved modelling of radiotherapy beams as they pass through normal lung tissue.^44^ In addition, tumour motion can be reliably visualised by the use of technologies, such as four-dimensional CT scanning during the radiotherapy planning stage.^45^ Finally, during treatment delivery, the accuracy of patient positioning and tumour localisation has improved dramatically with the use of image guidance such as cone beam CT (CBCT) prior to each treatment.^46,47^ Hence, the radiotherapy treatment delivered to patients is considerably different from that received a decade ago.^48^

A retrospective review of 409 NSCLC patients treated at the MD Anderson Cancer Center compared rates of pneumonitis in patients treated with three-dimensional conformal radiotherapy (3D-CRT) or IMRT.^49^ Whilst the patients treated with IMRT had more risk factors for the development of pneumonitis, more advanced disease, poorer PS and larger median gross tumour volume, the rates of grade ≥3 treatment-related pneumonitis at 1 year were significantly lower than patients treated with 3D-CRT (8% vs. 32%, respectively), possibly because of lower V20.^50^ Similarly in the RTOG 0617 study, the rate of grade ≥3 pneumonitis was twofold lower among patients treated with IMRT (3.5%) versus 3D-CRT (7.9%), despite patients receiving IMRT having more advanced disease and larger treatment volume to lung ratios compared with those treated with 3D-CRT.^51^ In addition, IMRT was associated with improved quality of life at 12 months.^52^

Quality assurance of radiotherapy plans with peer review is important in improving outcomes. In a meta-analysis of eight clinical trials with built-in quality assurance, including two studies in lung cancer patients, protocol deviations in radiotherapy delivery were associated with increased risks of treatment failure and overall mortality.^53^ In a United Kingdom (UK) study, 22% of radiotherapy treatment plans were changed with the use of peer review, with most of these changes being for the clinical target volume.^54^

## Optimisation of supportive care

Although often not formally evaluated within the context of clinical trials, there are a number of simple measures in terms of treatment and pathways that may improve outcomes. With aggressive symptomatic interventions, such as opioids, antacids, prophylactic antifungals and/or nutritional support, few patients require treatment breaks because of oesophagitis.^24^ Frequent assessment for dysphagia and early analgesia, if oesophagitis is developing, may help maintain treatment quality of life and preserve nutritional status; it has been reported that up to 67% of patients will need prescriptions for oesophagitis-associated pain.^55^ Patients receiving higher doses of cisplatin should receive antiemetics with a 5-HT_3_ and NK-1 antagonists. The importance of keeping patients well hydrated was demonstrated in a study assessing daily hydration with cisplatin, which showed lower renal dysfunction, improved treatment adherence and less oesophagitis in patients who were well hydrated.^56^

## Optimisation of nutrition

Poor nutrition in patients with lung cancer may be multi-factorial and related to the impact of previous poor or unbalanced intake, cancer-related cachexia and sarcopenia and the impact of treatment. In metastatic lung cancer, not only are cachexia and sarcopenia associated with poor survival, but also with poor tolerability and outcomes from therapy.^57,58^ The rates of malnutrition in advanced lung cancer are high, and may be as high as 69%,^59^ but in earlier-stage disease, even before treatment, many patients have experienced significant weight loss (20% of patients in one series).^60^ Significant weight loss (>5% of body mass) during cCRT for NSCLC is common (17% during the first 3 weeks in the publication by Sanders et al.,^60^ with 59% of patients experiencing some weight loss^61^), and was found to be associated with a poor prognosis.^61^ Unsurprisingly, radiotherapy dose to the oesophagus appears to be a strong predictor of weight loss during therapy as acute oesophagitis will have a detrimental impact on oral intake.^59^

Detection of patients who are already cachectic or sarcopenic is relatively simple using tools, such as nutrition-impact scales, grip strength and assessment of muscle bulk on staging CT scans.^62,63^ Evidence as to the appropriate dietary supplementation and/or dietary counselling for patients receiving CRT is lacking, with a recent meta-analysis across tumour types, stages and treatments, suggesting improvements in body weight when patients received polyunsaturated fatty acid-based supplements.^64^ A pilot study of intensive dietary counselling in 24 patients receiving cCRT or radiotherapy alone suggested beneficial outcomes for nutrition and quality-of-life endpoints, but was underpowered for significance.^65^ We suggest that patients should be monitored closely for oral intake and weight loss with weekly reviews during therapy and until resolution of toxicity. Patients who are already malnourished and receiving significant doses to the oesophagus should be counselled as to the additional risks and have early dietician intervention.

## Smoking cessation and optimisation of respiratory function

The vast majority of lung cancers are related to tobacco smoking, and some studies show that 24–60% of patients will be current smokers at the time of their lung cancer diagnosis.^66^ Patients who continue to smoke during radiotherapy will have accelerated reductions in lung function and worsening of existing respiratory illnesses, particularly chronic obstructive pulmonary disease compared with non-smokers.^67^ For patients who smoke, it will be significantly more difficult to achieve optimisation of respiratory function and therefore could decrease the treatment options available. There are many benefits of smoking cessation, including reduced risk of further disease, increased survival, increased efficiency of chemotherapy agents^66^ and increased quality of life. In addition, continued tobacco smoking after a diagnosis of lung cancer is associated with an increased risk of developing further synchronous primary tumours.^66^

Patients who have lung cancer and are smokers commonly have an increased psychological burden and experience stress due to the perceived opinions of society of their lifestyle choices and diagnosis.^68^ This may result in their quality of life being negatively affected. Furthermore, the high stress situation of a diagnosis of advanced lung cancer may make abstinence very difficult, and pharmacological and psychological intervention may be required.^69^

Most studies of smoking cessation have concentrated on earlier-stage NSCLC. A systematic review in this setting suggested that continued smoking in patients with early lung cancer was associated with increased recurrence and all-cause mortality;^70^ the risk of dying was almost tripled with continued tobacco smoking.^70^ However, even simple telephone contact may lead to some patients with lung cancer to stop smoking, and has been reported to be associated with improved survival.^71^

## Recommendations for chemoradiotherapy with immune- checkpoint inhibitors in patients with stage III NSCLC

As immunotherapies have been licensed to treat patients with stage IV NSCLC for several years, we can use the experience gained in this patient population when treating patients with stage III disease. The most important factors to consider are those related to treatment-related toxicities, in particular pulmonary AEs. Overall, serious immune-related toxicities are quite rare. Multiple guidelines exist for the management of immunotherapy toxicities, including a comprehensive guide from the European Society of Medical Oncologists.^72^ Given the variety of side effects that can be experienced, advising patients and family practitioners as to the symptoms that should prompt urgent review can be challenging, but should include changes in respiratory symptoms, diarrhoea, abdominal pain, severe joint or muscle pain, fatigue or confusion. Patients should be asked about these symptoms and visits they have had with other health professionals at each oncology attendance, and they should be monitored with full blood count, blood urea and electrolyte studies, liver function tests and thyroid function at every visit.^73^ Early recognition and close monitoring of these toxicities and cross-collaboration with disease specialists can improve clinical outcomes while minimising harm to patients. Cross-sectional imaging is recommended at the end of CRT to establish early response, and that the criteria for subsequent immunotherapy have been met, and then regularly during therapy to confirm response (as full response may take some time to establish^74^) and to assess for any radiological evidence of pneumonitis^73^ (our current practice is every 3 months).

The most common symptom that may raise concern following immunotherapy is the possibility of pneumonitis, which was observed in 24.8% of the patients in the placebo arm in PACIFIC.^30^ To ensure prompt diagnosis and management of pneumonitis, frequent monitoring of, and patient education on, signs or symptoms of possible pneumonitis, such as new or worsening cough, wheezing, dyspnoea or fatigue, is essential. The differential diagnosis is wide with the most common aetiology being pneumonitis (secondary to radiotherapy or immunotherapy), but also includes recurrent cancer, pneumonia, atypical infections (including *Mycoplasma*, *Mycobacteria*, *Legionella* and *Pneumocystis jiroveci*), pulmonary emboli and pulmonary oedema. Determining the cause is important not only for immediate management, but also for decisions as to reinstating immunotherapy on recovery. All patients should undergo urgent imaging with high-resolution CT of the chest, and those with radiographic and/or clinical evidence of pneumonitis should be started on high-dose steroids^72^ and referred to a pulmonary specialist.^73^ Lung changes that are restricted to the radiotherapy fields are more likely to be related to radiotherapy compared with the more diffuse changes that are observed with immunotherapy pneumonitis, and bronchoscopy may be used to help rule out infection.^75^

## Conclusion

**Fig. 2:**
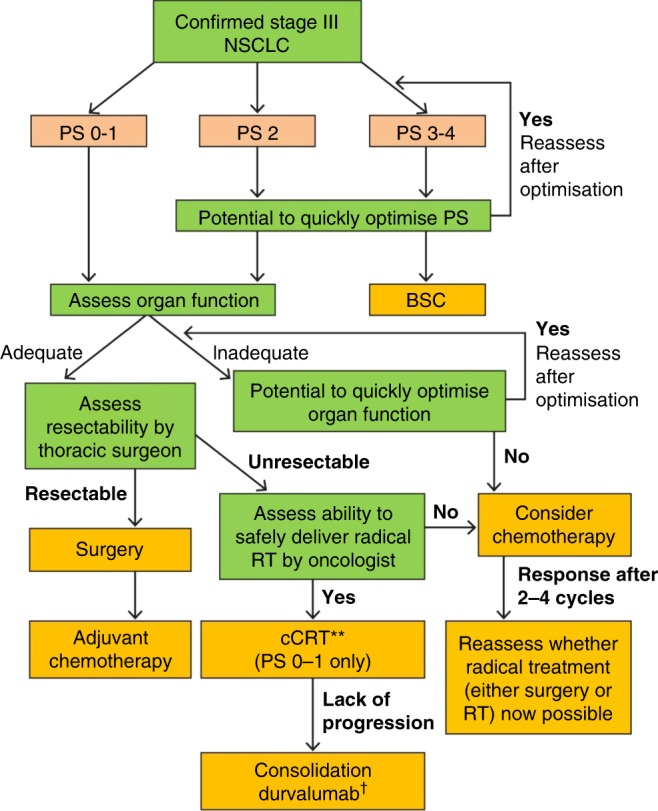
Algorithm of the potential approach to treatment with curative- intent decisions in patients with stage III NSCLC. This figure was created by the author, using guidance from refs. ^1,2,77^ Stage III NSCLC is a heterogeneous disease with both tumour extent and patient fitness being important factors in advising the optimum treatment. A potential approach to treatment decisions is outlined; however, patient wishes, local guidelines and the capabilities of the treating teams should also be considered. In patients not suitable for radical treatment, frequent re-evaluation of both tumour extent and fitness is recommended to determine if a window of opportunity has emerged for the initiation of such treatments. *Platinum-based CRT. ^†^In patients whose tumours express PD-L1 on at least 1% of tumour cells and whose disease has not progressed after platinum-based chemoradiation. BSC best supportive care, CRT chemoradiotherapy, NSCLC non-small-cell lung cancer, PS performance status, RT radiotherapy.

Stage III NSCLC is a heterogeneous disease with both tumour extent and patient fitness being important factors in advising the optimum treatment. A potential approach to treatment decisions is outlined in Fig. 2; however, patient wishes, local guidelines and the capabilities of the treating teams should also be taken into account. In patients not suitable for radical treatment, frequent re-evaluation of both tumour extent and fitness is recommended to determine if a window of opportunity has emerged for the initiation of such treatments. An experienced multidisciplinary team is required to assess and safely deliver CRT treatment in patients with stage III NSCLC to ensure maximum access for this potentially curative therapy. This will be even more important as we add additional therapies to improve outcomes following completion of cCRT, such as consolidation immune-checkpoint inhibitors (in patients who are responding, have a good PS [PS 0–1] and have no contraindications to immunotherapy). A diagnostic workup and assessment of patient fitness is required to determine optimal treatments, despite the potential complexity of the pathway. Along with the risks of metastases, tumour growth will lead to increased treatment volumes, resulting in greater treatment-associated toxicity, and more problems with treatment delivery. Combined clinics of surgeons and clinical oncologists may lead to faster decision-making for the most appropriate treatment modality for individual patients with radically treatable stage III NSCLC. In addition, frequent review by oncologists and lung nurses, with input from dieticians, palliative care professionals, respiratory and care-of-the-elderly physicians during treatment, may also help in safer and more effective treatment delivery. A diagnosis of lung cancer may be a ‘teachable moment’ for both patients and their relatives to encourage smoking cessation,^76^ which could help improve the long-term outcomes of patients diagnosed with stage III NSCLC treated with cCRT.

## Data Availability

Not applicable.

## Appendix

**Prescribing information**

IMFINZI^®^ ▼(durvalumab) 50 mg/ml solution for infusion

https://medicines.astrazeneca.co.uk/content/dam/multibrand/uk/en/prescribinginformation/imfinzi-pi.pdf
